# Polyvinylpyrrolidone as additive for perovskite solar cells with water and isopropanol as solvents

**DOI:** 10.3762/bjnano.10.228

**Published:** 2019-12-05

**Authors:** Chen Du, Shuo Wang, Xu Miao, Wenhai Sun, Yu Zhu, Chengyan Wang, Ruixin Ma

**Affiliations:** 1 School of Metallurgical and Ecological Engineering, University of Science and Technology Beijing, Beijing 100083, PR China; 2 Beijing Key Laboratory of Rare and Precious Metals Green Recycling and Extraction, University of Science and Technology Beijing, Beijing 100083, PR China; 3 Beijing Key Laboratory of Special Melting and Preparation of High-End Metal Materials, Beijing 100083, PR China

**Keywords:** additive, lead nitrate aqueous solution, low-toxicity process, perovskite solar cells, polyvinylpyrrolidone

## Abstract

The recent years have witnessed a fast-paced development of perovskite solar cells (PSCs). Unfortunately, the vast majority of PSCs relies on the use of highly polar aprotic solvents during the preparation process, such as dimethylformamide (DMF), which is toxic and detrimental to both humans and the environment. Here, we describe the preparation of PSCs under ambient conditions from an aqueous solution of lead nitrate, to which polyvinylpyrrolidone (PVP) was added in order to enhance the photoelectric performance of the PSCs. By a combination of SEM, EIS, PL and UV spectroscopy and other characterization approaches, we show that the PVP additive is effective in inhibiting carrier recombination, enhancing composite resistance and reducing film defects. Ultimately, we achieved an outstanding photoelectric performance of the PVP-doped PSCs shown by a power conversion efficiency (PCE) of 15.19% and an average steady-state PCE of 14.55% under AM 1.5G simulated solar irradiation with a shadow mask of 0.1 cm^2^. The PCE continued to be over 80% of the initial PCE after 60 days of storage. FInally, the introduced PVP-doped PSCs present a low-cost and low-toxicity way to commercialize perovskite solar cells.

## Introduction

Due to the restrictions imposed by resources and the environment on the use of fossil fuels, in recent years, clean renewable energy technologies have received increasing attention, which has also driven the growth of the photovoltaic industry [1–4]. The remarkable light absorption capacity [5] and the tunable band gap [6] of inorganic–organic lead halide perovskite crystals make them suitable for the production of organic semiconductors [7], photodetectors [8], and photovoltaics [5]. In 2009, Kojima et al. achieved a breakthrough in using mesoporous TiO_2_ as photoanode and CH_3_NH_3_PbX_3_ (X = I, Br) as light absorbing material to derive perovskite dye-sensitized solar cells with conversion efficiencies of 3.81% (X = I) and 3.13% (X = Br). However, the corrosion of the perovskite material used in the electrolyte results in a poor stability of the devices [5]. In July 2018, a world record was set by Jingbi You et al., who presented perovskite solar cells with an efficiency of 23.3% [9].

Perovskite solar cells can be flexibly constructed from crystals of different components with different groups, which represents a vital advantage [2,10–14]. As one of the most popular perovskite materials, MAPbI_3_ can be generated from various lead-containing precursors, such as lead iodide (PbI_2_) [3,15–16], lead acetate [17–18], lead iodide [19], and lead chloride [10,20]. Most of the preparations involve highly polar aprotic solvents such as dimethylformamide (DMF). DMF is a toxic solvent with a clinical link to liver disease, although the pathology remains unknown [21–22]. Therefore, to prepare high-efficiency PSCs by sequential deposition, water-based lead precursors and lead nitrate (Pb(NO_3_)_2_) were used as raw materials by Tsung-Yu Hsieh et al., with the highest PCE of PSCs being 15.11% [23–24]. A procedure to synthesize perovskite absorbers using only water and isopropanol as solvents was developed by Sveinbjörnsson and co-workers. Also, by using the perovskite composition Cs_0.1_FA_0.9_Pb(I_0.83_Br_0.17_)_3,_ they obtained an average solar cell power conversion efficiency of 13.0% [25]. Through these works, the use of toxic solvents such as DMF in the preparation process is avoided, which could reduce the potential damage caused by perovskite solar cells to the environment. However, the photoelectric effect of perovskite solar cells prepared from water-based lead nitrate remains less than satisfactory.

A common method to improve the photoelectric performance of perovskite solar cells is adding organic polymers. Jianfeng Li et al. [26–27] and Zezhou Liang et al. [28] both improved the photoelectric performance of perovskite solar cells by adding organic polymers. As a non-ionic polymer compound, polyvinylpyrrolidone (PVP) shows excellent solubility and physiological compatibility and is extensively applied in medicine, food, and cosmetics, which are closely related to the health of people [29]. PVP is commonly integrated in PSCs, which is primarily due to the suitability of PVP as an additive to enhance the photoelectric performance. Using PVP as an interlayer between the [6,6]-phenyl-C_61_-butyric acid methyl ester (PCBM) electron transport layer (ETL) and the Ag cathode in a high-performance inverted planar heterojunction perovskite solar cell (iPSC), Pengcheng Zhou et al. managed to achieve a signifanct boost in efficiency [30]. The photovoltaic properties of inverted polymer solar cells using a PVP-modified indium tin oxide (ITO) layer as the electron-collecting electrode were reported on by J. W. Shim and co-workers [31].

In this paper, we describe the preparation of perovskite solar cells from an aqueous solution of lead nitrate as precursor under ambient conditions. In order to improve the photoelectric performance of the PSCs, PVP was added to the precursor solution. A combination of SEM, EIS, PL, UV, and other characterization methods show that PVP was effective in inhibiting the recombination of carriers, enhancing the composite resistance, reducing film defects, and boosting the photoelectric performance of the PSCs. With a PCE of 15.19% and an average steady-state PCE of 14.55% under AM 1.5G simulated solar irradiation with a shadow mask of 0.1 cm^2^, the best optoelectronic performance of the PVP-doped PSCs was achieved and it remained at over 80% of the initial value after 60 days of storage. In contrast to previous works in which PVP was used as additive, we avoid the use of DMF during preparation, and the whole process was carried out under ambient conditions. Thus, our preparation method involves less toxic substances and is more environmentally friendly. Compared with previous preparation methods using aqueous lead nitrate solution as a precursor, our method can improve the photovoltaic performance of solar cells, and the preparation method is relatively simple. Overall, it is a low-cost, low-toxicity preparation method to commercialize perovskite solar cells.

## Experimental

### Materials

Lead nitrate, polyvinylpyrrolidone, chlorobenzene and isopropanol were purchased from Aladdin. SnO_2_ colloid was purchased from Alfa Aesar. Methylammonium iodide (MAI), methylammonium chloride (MACl) and Spiro-OMeTAD were purchased from Xi’an Polymer Light Technology Corp. ITO glasses with a sheet resistance of 8 Ω/sq were also purchased from Xi’an Polymer Light Technology Corp. All chemicals were used as recieved.

### Device fabrication

Prior to spin-coating, the ITO glass substrate was cleaned sequentially with detergent, acetone, ethanol, deionized water, each for 15 min, then dried with a nitrogen flow, and then cleaned with UV/ozone for 20 min. A thin layer of SnO_2_ nanoparticles was spin-coated on the ITO substrate at 4000 rpm for 30 s and annealed at 150 °C for 10 min, then treated with UV/ozone for 20 min. The Pb(NO_3_)_2_ layer was prepared by spin-coating an aqueous Pb(NO_3_)_2_ solution (the concentration of Pb(NO_3_)_2_ was 1.0 M, doped with 0, 0.5, 1, 2, or 3 mg/mL PVP) at 4000 rpm for 20 s and annealed at 100 °C for 10 min. Then the film was submerged in the MAX solution (the concentration of the MAX solution was 40 mg/mL MAI and 10 mg/mL MACl) for 500 s to prepare the CH_3_NH_3_PbI_3_ layer, and the films were dried by spinning at 3000 rpm for 10 s and annealing at 120 °C for 10 min. Subsequently, the hole transport layer (HTL) was spin-coated on top of the CH_3_NH_3_PbI_3_ film using a Spiro-OMeTAD solution (the composition of the Spiro-OMeTAD solution was 72.3 mg Spiro-OMeTAD, 28.8 μL 4-*tert*-butylpyridine, 17.5 μL of lithium bis(trifluoromethanesulfonyl)imide solution (520 mg/mL in acetonitrile) and 1 mL chlorobenzene) at 4500 rpm for 20 s. The above preparation process was carried out under ambient conditions. Finally, the devices were completed by using thermal evaporation and deposited on an Au electrode.

### Characterization

Field-emission scanning electron microscopy (FESEM) images were obtained with a ZEISS SUPRA55. X-ray diffraction (XRD) patterns were collected with a SmartLab from Rigaku at 40 kV and 150 mA by using Cu Kα radiation (λ = 0.15405 nm). The photovoltaic performance of the PSCs was recorded using a Keithley 4200 source meter under one-sun AM 1.5G (100 mW·cm^−2^) illumination with a solar light simulator (Newport Oriel Sol3A Class AAA, 64023A Simulator), which was calibrated using a NREL standard Si solar cell. Electrochemical impedance spectroscopy (EIS) measurements were conducted by using an electrochemical workstation (CHI660d) (1 MHz to 100 Hz) and for fitting the Zview software was used. The UV–vis light absorption measurements were performed by using a spectrophotometer (Shimadzu UV-3101 PC). The external quantum efficiency (EQE) measurements were obtained on a Keithley 2000 multimeter as a function of the wavelength from 350 to 800 nm on the basis of a Spectral Products DK240 monochromator. Infrared (IR) spectroscopy was performed using a Fourier transform IR spectrometer (VERTEX80v, Bruker). Photoluminescence (PL) spectra and the fluorescence decay curves were recorded with a computer controlled, modular spectro- fluorimeter (FLS980, Edinburgh). The active area of the cell was 0.1 cm^2^. All samples were measured in air (25 °C).

## Results and Discussion

**Figure 1:**
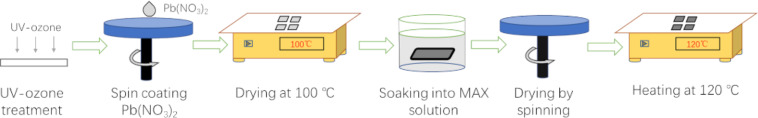
Fow chart of the preparation the PSCs.

Figure 1 illustrates how the PSCs were prepared. The PSCs of this study comprises a SnO_2_ electron transfer layer (ETL), a MAPbI_3_ coating, a Spiro-OMeTAD HTL as well as a gold electrode. In a first step, the perovskite layer was irradiated by UV/ozone. The following most critical point in the production of the perovskite layer was to spin the lead nitrate onto the SnO_2_ ETL. Then, the layer was introduced into a bath of MAX solution for 500 s, and finally dried by spinning and annealing at 120 °C for a period of 10 min.

**Figure 2:**
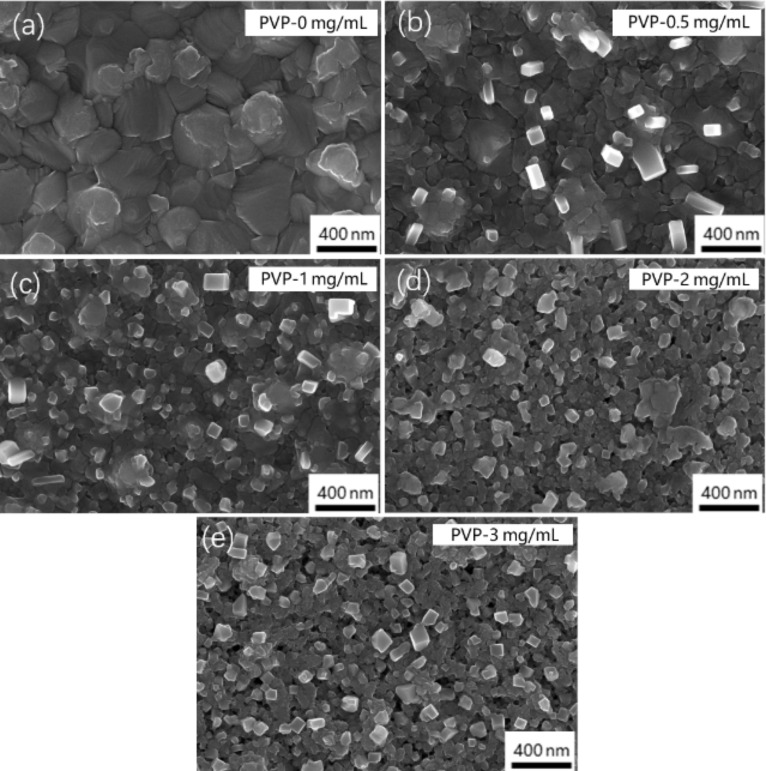
(a–e) SEM images of perovskite films prepared with different amounts of PVP additives: (a) PVP-0 mg/mL, (b) PVP-0.5 mg/mL, (c) PVP-1 mg/mL, (d) PVP-2 mg/mL, (e) PVP-3 mg/mL.

The SEM images of the perovskite films (Figure 2a–e) reveal that the surface of the perovskite films is dense and flat when the volume of the PVP additive is 0, 0.5, or 1 mg/mL. When the volume of the PVP additive reaches 2 or 3 mg/mL, the surface of the perovskite films gets rough with voids. All samples show small cubes on the surface, which are attributed to the surface crystallization of MAX residual after the immersion. The photoelectric performance of the PSCs is unaffected by the small cubes.

**Figure 3:**
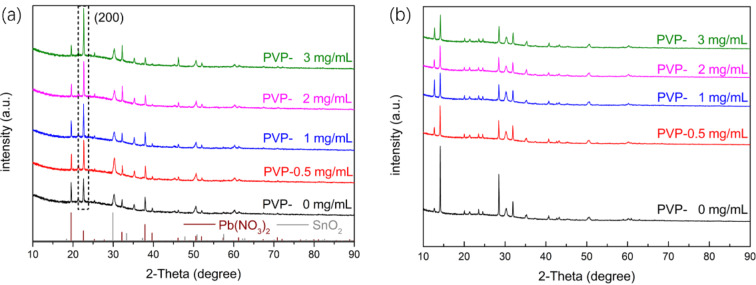
(a) XRD patterns of the Pb(NO_3_)_2_ films with different amounts of PVP additive. (b) XRD patterns of the perovskite films with different amounts of PVP additive.

The XRD patterns of the Pb(NO_3_)_2_ films coated on the SnO_2_ ETL are presented in Figure 3a. Pb(NO_3_)_2_ contains no impurities. There is only the SnO_2_ peak of the ETL. As the amount of PVP increases, the intensity of the Pb(NO_3_)_2_ (200) peaks increases indicating a preferential growth of the Pb(NO_3_)_2_ films. Figure 3b reveals the occurrence of typical solution-treated perovskite MAPbI_3_ diffraction peaks and no occurrence of impurity peaks [32].

**Figure 4:**
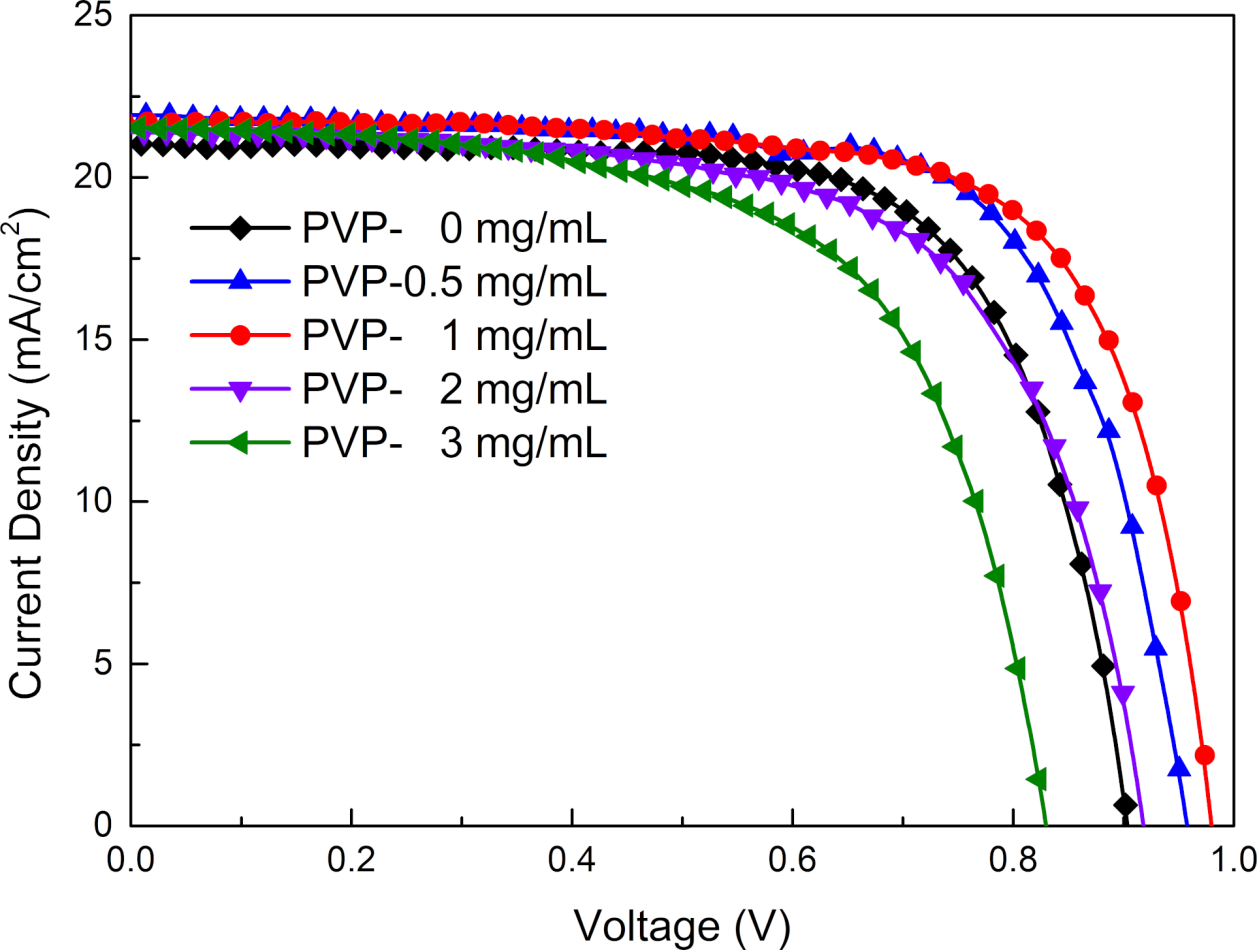
The current density–voltage curves of the perovskite solar cells fabricated under different conditions.

Figure 4 shows the photovoltaic performance of the perovskite solar cells prepared with different amounts of PVP additive. The measured parameters are listed in Table 1. The open-circuit voltage (*V*_oc_) is shown to be affected massively, not only by the relative position of the quasi-Fermi level in the contacted perovskite and electron transfer material, but also by the defect-induced recombination in the electron transport channels [33]. It is clear that perovskite solar cells using a PVP-containing aqueous lead nitrate precursor solution will lead to an increase in *V*_oc_. The *V*_oc_ of the PSCs increases from 0.90 to 0.98 V and the PCE of the PSCs is found to increase from 13.33% to 15.19%. Through the combination of PVP and lead nitrate, the morphology of the perovskite film was improved, film defects were reduced, the interface contact was improved, and the carrier recombination was reduced. This improves the photoelectric performance of perovskite solar cells (including *V*_oc_, *J*_sc_, and FF), and is mainly reflected in the improvement of *V*_oc_. It is worth noting that the addition of 2 or 3 mg/mL PVP is accompanied by a decline of the photovoltaic performance of the PSCs. This is attributed to the appearance of voids on the surface of the perovskite layer, which results in a higher recombination rate of the carriers [34].

**Table 1:** Parameters of the PSCs fabricated under different conditions.

sample	*J*_sc_ (mA/cm^2^)	*V*_oc_ (V)	FF	PCE (%)

PVP-0 mg/mL	21.03	0.90	0.70	13.33
PVP-0.5 mg/mL	21.93	0.96	0.71	14.81
PVP-1 mg/mL	21.72	0.98	0.71	15.19
PVP-2 mg/mL	21.40	0.92	0.66	12.89
PVP-3 mg/mL	21.53	0.83	0.63	11.25

**Figure 5:**
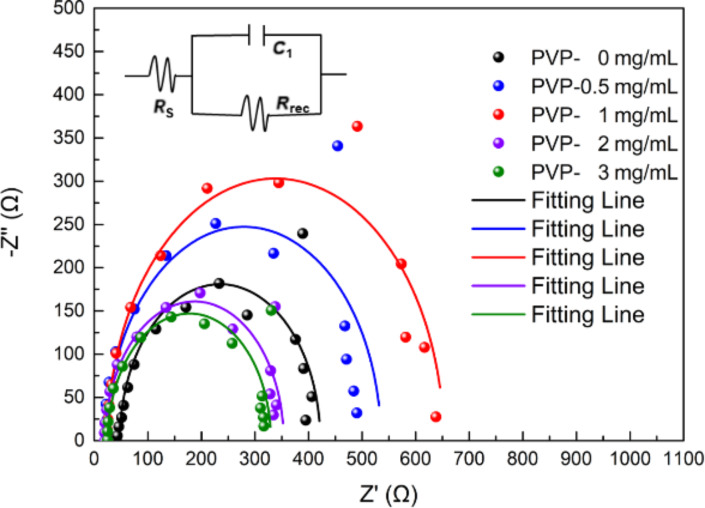
Nyquist plot of the PSCs measured within the frequency range from 100 Hz to 1 MHz under AM 1.5G irradiation at a direct current bias of 0 V. Symbols are experimental data and solid lines correspond to the fits using the equivalent circuit (inset).

Figure 5 shows electrical impedance spectroscopy (EIS) measurements at a voltage bias of 0 V under one-sun light intensity with the test frequency ranging from 0.1 MHz to 100 Hz. The fit results are presented in Table 2. In the circuit model, the resistors derived from metal contacts, compound resistors, and chemical capacitors are denoted by the symbols *R*_S_, *R*_rec_, and *C*_1_, respectively. As the cell structure is uniform, the difference of the *R*_S_ values between the five devices is reduced. A higher level of *R*_rec_ relates to a larger arcs in the figure, which suggests a smaller number of composite centers in the solar cell [35]. When the amount of PVP additive is 0.5 and 1 mg/mL, the *R*_rec_ value is greater than that of devices without PVP. In contrast, when 2 or 3 mg/mL PVP is added, the *R*_rec_ value is lower than that of devices without added PVP. This is because the surface of the perovskite film gets rough with voids, which results in a higher recombination rate of the carriers. This is consistent with the variation of the photoelectric performance of the five devices. The *R*_S_ value is related to the cell structure. Since the five kinds of devices are similar in structure, their *R*_S_ values are relatively close. The slower the carrier recombination in the solar cell, the larger the value of the *R*_rec_. After appropriate PVP treatment, the devices have more satisfactory interface contact and slower carrier recombination and, thus, a higher *R*_rec_ value, which in turn leads to a rise in *V*_oc_.

**Table 2:** Fit results of the Nyquist plot.

sample	*R*_S_ (Ω)	error rate (%)	*R*_rec_ (Ω)	error rate (%)

PVP-0 mg/mL	47.86	6.47	373.9	5.45
PVP-0.5 mg/mL	23.37	10.25	511.4	6.67
PVP-1 mg/mL	24.93	6.82	626.7	4.75
PVP-2 mg/mL	20.72	10.42	332.6	6.61
PVP-3 mg/mL	25.57	7.85	304.1	5.24

**Figure 6:**
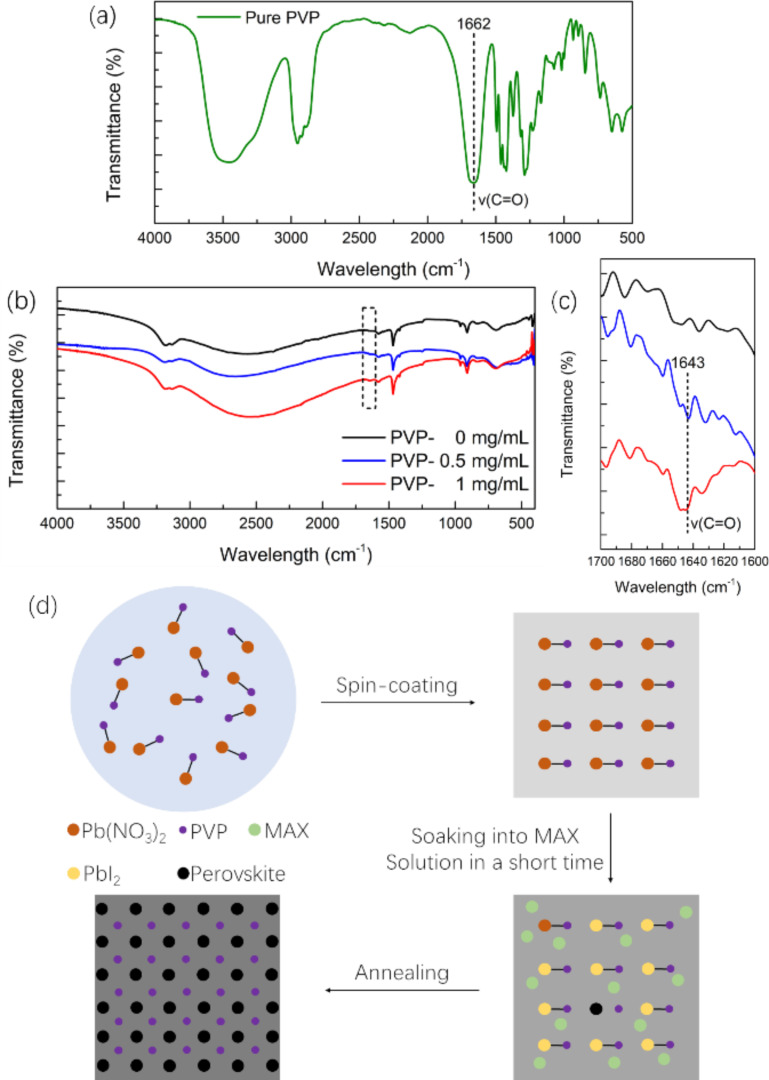
(a) Infrared spectrum of pure PVP over the full scan range. (b, c) Infrared spectra of the perovskite films; (b) full scan range and (c) expanded fingerprint region from 1700 to 1600 cm^−1^. (d) Schematic diagram and schematic reaction process of the deposition of perovskite films.

In order to figure out the role of PVP in the perovskite formation process, IR spectroscopy studies were conducted, with the IR spectrum results of pure PVP shown in Figure 6a. The position of the C=O stretching vibration in pure PVP is 1662 cm^−1^, in line with the results obtained by Bo Li and co-workers [36]. The IR spectra of the perovskite films with three different amounts of PVP additive are given in Figure 6b. After the addition of small amounts of PVP, the C=O stretching vibration is insignificant. Figure 6c shows an enlargement of the position of the C=O stretch of Figure 6b (dashed box). The C=O stretching vibration of the perovskite films appears at lower wavenumbers than that of pure PVP, because the formation of the adduct causes the strength of the carbon–oxygen bond to be reduced. Based on the analysis conducted above, the main reaction process is illustrated in Figure 6d. Firstly, Pb(NO_3_)_2_ is combined with PVP in aqueous solution. Following spin-coating, a film of a Pb-PVP phase forms. During the immersion in MAX solution, PVP combines with Pb(NO_3_)_2_ to decelerate the reaction between Pb(NO_3_)_2_ and MAX, thus making the perovskite film less defective, which is thought to be conducive to the performance of the PSCs [34].

**Figure 7:**
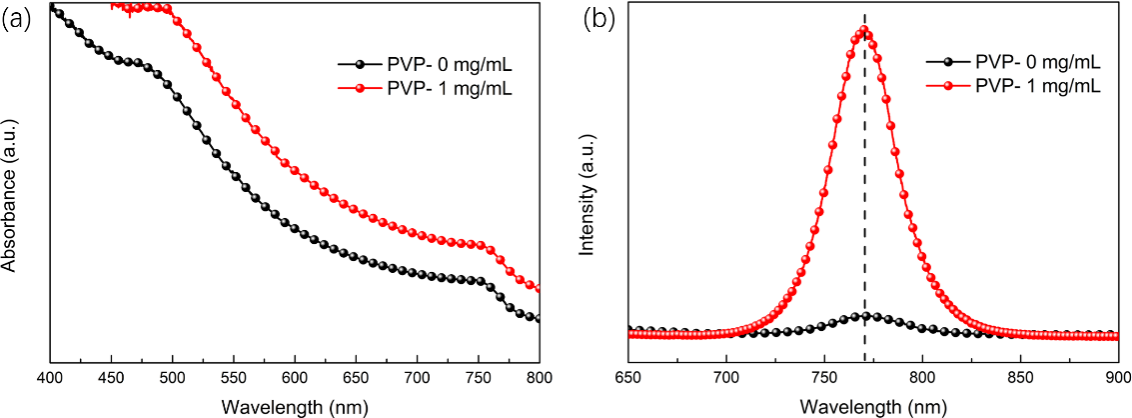
(a) UV–vis measurement of the film without PVP additive and the film with PVP additive of 1 mg/mL. (b) The steady-state photoluminescence curves with an emission maximum at 760 nm were obtained upon excitation at 445 nm.

UV-vis measurements were carried out to determine the influence of PVP on the optical properties of the perovskite films (Figure 7a). In comparison to the film without PVP, the film with added PVP displays a better capability of absorption in the visible-light range, which is due to PVP triggering a slight red shift in the absorption edge. This is attributed to a smaller number of defects and possibly less reflection [37]. To explore the emission characteristics of the perovskite crystal MAPbI_3_, a quenching steady-state PL spectroscopy study was performed (Figure 7b). The preparation of all samples was made on m-SnO_2_/ITO substrates. As PVP was added, there was a noticeable increase in the intensity of the emission peak of perovskite and a slight decline in the FWHM of the final product, which indicates a reduction in surface defects.

**Figure 8:**
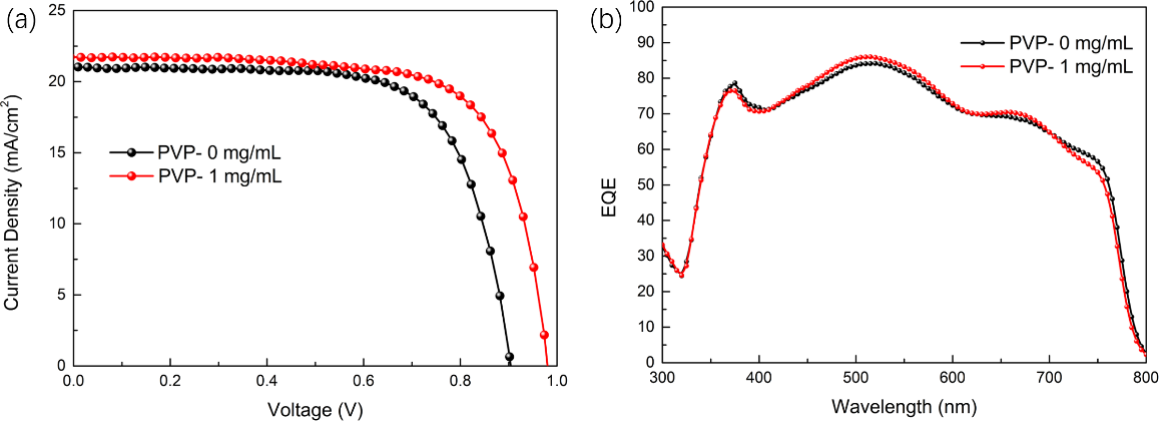
(a) The current density–voltage curves of PSCs (b) EQE spectra of PSCs.

The *J*–*V* curves of the films with optimal amount of PVP additive and without PVP additive are shown in Figure 8a. The preparation of a perovskite solar cell using a PVP-containing aqueous lead nitrate precursor solution is found to be effective in increasing the *V*_oc_. The short-circuit photocurrent density (*J*_sc_) exhibits no significant difference, which has also been manifested by measuring the external quantum efficiency (EQE) of the two samples over the entire wavelength range from 350 to 800 nm (Figure 8b). The EQE of the two samples is nearly identical, which also indicates that their *J*_sc_ is almost identical.

**Figure 9:**
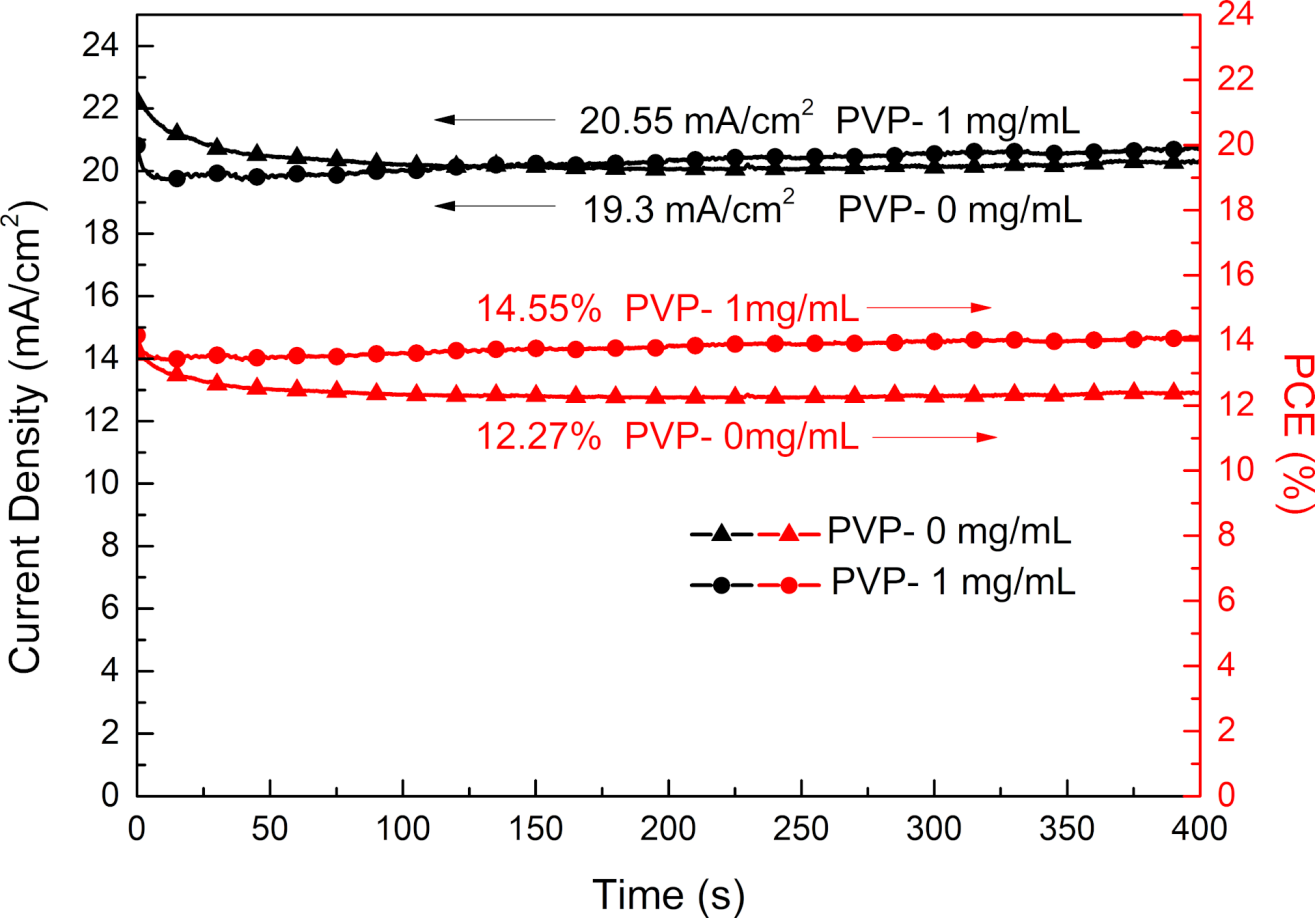
The stable output current density and PCE at the max power point of PSCs.

The stable PCEs measured at the maximum power point of the films with optimal amount of PVP additive and without PVP additive are presented in Figure 9, which reveals a steady-state current density of 20.55 mA/cm^2^, along with an efficiency of 14.55% after 400 s operation at a bias voltage of 0.71 V for the device based on the optimum amount of PVP additive. Besides, for the device without PVP additive, the steady-state current density is shown to be 19.3 mA/cm^2^ at a bias voltage of 0.64 V, and the efficiency is 12.27%. This demonstrate clearly that the PVP as an additive to the aqueous solution of Pb(NO_3_)_2_ is genuinely effective in boosting the photoelectric performance of the device.

**Figure 10:**
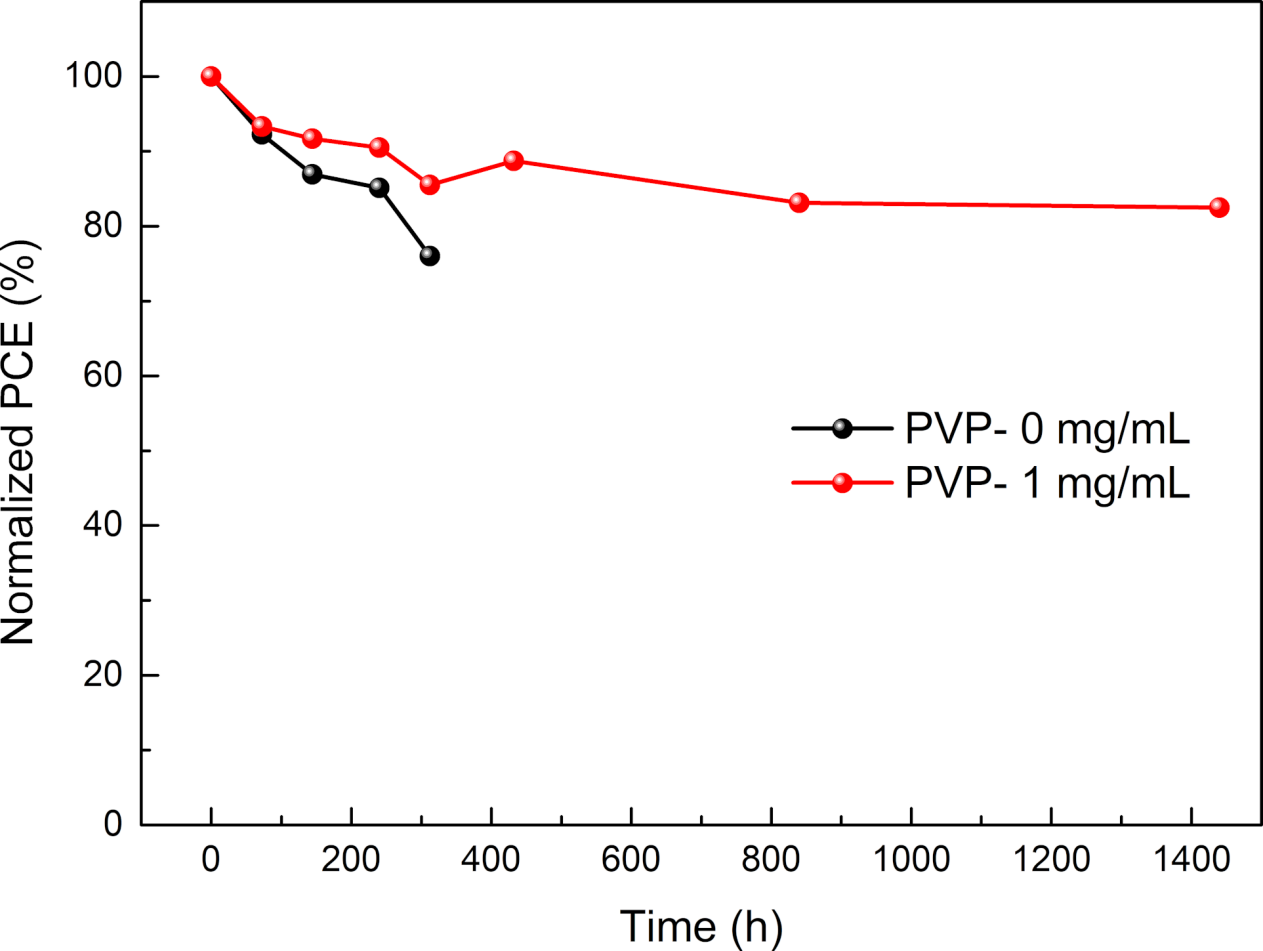
Long-term stability of solar cells over 60 d. All devices were stored in ambient environment (humidity less than 10%, room temperature).

In addition, we studied the long-term stability of the PSCs in ambient environment (humidity less than 10%, room temperature), and the results are presented in Figure 10. The cells with the optimum amount of added PVP exhibit excellent stability in ambient air. The devices retained over 80% of the initial PCE after 60 days. In comparison, the devices without PVP additive preserved 76% of the initial PCE after as little as 400 h. The higher stability in ambient air is attributed to the lower number of surface defects [38].

## Conclusion

The photovoltaic performance of PSCs with an aqueous lead nitrate solution as the precursor was enhanced through the addition of PVP. The PVP additive influenced the crystallization process. The optimum amount of PVP additive was 1 mg/mL, which was effective in reducing the number of defects of the perovskite film leading to better charge transfer and separation on the interface. Besides, the film exhibited a stronger absorption in the visible-light range. The best performance of PSCs with PVP is characterized by a PCE of 15.19% and an average steady-state PCE of 14.55%. The solar cell with added PVP displayed a better stability in ambient air. Thus, a low-cost and low-toxicity method to commercialize perovskite solar cells has been presented.
